# Potential neutralizing antibodies discovered for novel corona virus using machine learning

**DOI:** 10.1038/s41598-021-84637-4

**Published:** 2021-03-04

**Authors:** Rishikesh Magar, Prakarsh Yadav, Amir Barati Farimani

**Affiliations:** 1 Department of Mechanical Engineering, Carnegie Mellon University, Pittsburgh, PA 15213 USA; 2 Department of Biomedical Engineering, Carnegie Mellon University, Pittsburgh, PA 15213 USA; 3 Machine Learning Department, School of Computer Science, Carnegie Mellon University, Pittsburgh, PA 15213 USA

**Keywords:** Proteins, Computational models, Machine learning, Biophysics, Computational biophysics, Cheminformatics

## Abstract

The fast and untraceable virus mutations take lives of thousands of people before the immune system can produce the inhibitory antibody. The recent outbreak of COVID-19 infected and killed thousands of people in the world. Rapid methods in finding peptides or antibody sequences that can inhibit the viral epitopes of SARS-CoV-2 will save the life of thousands. To predict neutralizing antibodies for SARS-CoV-2 in a high-throughput manner, in this paper, we use different machine learning (ML) model to predict the possible inhibitory synthetic antibodies for SARS-CoV-2. We collected 1933 virus-antibody sequences and their clinical patient neutralization response and trained an ML model to predict the antibody response. Using graph featurization with variety of ML methods, like XGBoost, Random Forest, Multilayered Perceptron, Support Vector Machine and Logistic Regression, we screened thousands of hypothetical antibody sequences and found nine stable antibodies that potentially inhibit SARS-CoV-2. We combined bioinformatics, structural biology, and Molecular Dynamics (MD) simulations to verify the stability of the candidate antibodies that can inhibit SARS-CoV-2.

## Introduction

The biomolecular process for recognition and neutralization of viral particles is through the process of viral antigen presentation and recruitment of appropriate B cells to synthesize the neutralizing antibodies^1^. Theoretically, this process allows the immune system to stop any viral invasion, but this response is slow and often requires days, even weeks before adequate immune response can be achieved^2,3^. This poses a challenging question: can the process of antibody discovery be computationally accelerated to counter highly infective viral diseases?

The general paradigm of computational antibody design revolves around doing complex Molecular dynamics (MD) simulations that are computationally expensive. The computational expense of MD simulations makes them inaccessible in scenarios like global pandemic when rapid solutions are needed that can be reliable and accurate. Thus, it is imperative to design and develop techniques that can aid the computational antibody discovery process. With the rapid expansion of available biological data, such as DNA/protein sequences and structures^4^, machine learning (ML) approaches have been increasing used in modelling and predicting biological phenomenon^5,6^. Given sufficient training data, ML can be used to learn a mapping between the viral epitope and effectiveness of its complementary antibody. Once such mapping is learnt, it can be used to predict potentially neutralizing antibody for a given viral sequence enabling us to design novel antibodies^7^. Thus, enabling ML models to be used for high throughput screening of antibody sequences which is faster than traditional methods of computational protein design using MD simulations.

ML can learn the complex antigen–antibody interactions much faster than human immune system. This allows rapid generation of a library of synthetic inhibitory antibodies bridge, which can overcome the latency between viral infection and human immune system response. This bridge can potentially save the life of many people during the outbreak of novel viruses for which we lack treatment. One such instance is the spread of coronavirus disease (COVID-19)^8^.

With incredibly high infectivity and mortality rate, COVID-19 has become a global scare^9,10^. Although the vaccines against COVID-19 are now available but there are no proven therapeutics, such as antibody serum, to aid the suffering patients^2,9,11–18^. Vaccines are a preventive measure to stop the spread of COVID-19, but do not have a therapeutic effect if a patient has been infected by SARS-CoV-2. Antibody serum based therapies can help patients after they have been infected by the SARS-CoV-2. Only viable treatment at the moment is symptomatic and there is a desperate need for developing therapeutics to counter COVID-19. Recently, the proteomics sequences of ‘WH-Human 1’ coronavirus became available through Metagenomic RNA sequencing of a patient in Wuhan^4,19^. WH-Human 1 is 89.1% similar to a group of SARS-like coronaviruses^4^. With the availability of this sequence, it is possible to find potential inhibitory antibodies by scanning thousands of antibody sequences and discovering the neutralizing ones^20–22^. However, this requires very expensive and time-consuming experimentation to discover the inhibitory responses to SARS-CoV-2 in a timely manner. In addition, computational and physics-based models require the bound crystal structure of antibody-antigen complex, however; only a few of these structures have become available^23–26^. Moreover, in case of SARS-CoV-2, the complex of viral antigen and neutralizing antibody is not available to-date^27,28^. Due to the lack of availability of structural data we aimed to develop an ML model which leverages the information in the antibody-antigen sequences rather than the structures to predict the potential neutralizing antibodies^29^.

In this paper, we have collected a dataset comprised of antibody-antigen sequences of variety of viruses including HIV, Influenza, Dengue, SARS, Ebola, Hepatitis, etc. with their patient clinical/biochemical IC_50_ data. Using this dataset (we call it VirusNet), we trained and benchmarked different shallow and deep ML models and selected the best performing model, to predict a set of potentially neutralizing antibodies. Based on SARS 2006 neutralizing antibody scaffold^30^, we created thousands of antibody candidates by mutation and screened them with our best performing ML model to find the potentially neutralizing antibodies. Finally, molecular dynamics (MD) simulations were performed on the neutralizing candidates to check their structural stability. We predict nine structures that were stable over the course of simulation and are potential neutralizing antibodies for SARS-CoV-2. In addition, we interpreted the ML method to understand what alterations in the sequence of binding region of the antibody would most effectively counter the viral mutation(s) and restore the ability of the antibody to bind to the virus^31^. This information is critical in terms of antibody design and engineering in order to reduce the dimension of combinatoric mutations needed to find a neutralizing antibody.

This work highlights the merits of leveraging an ML based method for high-throughput discovery of neutralizing antibodies for viruses where only the sequences of viral coat protein-antibody pairs can be obtained. Moreover, this work also proposes a recipe for computational antibody design using ML approaches to work concurrently with the traditional molecular dynamics simulations-based approaches in order to augment each other. Through our computational approach we are able to leverage ML techniques to computationally design antibodies and also take advantage of accepted paradigm of molecular dynamics to validate our ML based approach.

## Methods

### Dataset

The VirusNet dataset consists of 1933 samples spanning over 15 different types of viruses. Majority of the data in the training set is composed of HIV antibody-antigen complex as it widely studied and readily available. Most of the samples for the HIV training set were obtained from the Compile, Analyze and Tally NAb panels (CATNAP) database from the Los Alamos National Laboratory (LANL)^32,33^. From CATNAP, data was collected for monoclonal antibodies, 2F5, 4E10 and 10E8, which bind with GP41^34–36^. Using CATNAP’s functionality for identifying epitope alignment, we selected FASTA sequence of the antigen corresponding to the site of alignment, in the antibody. We generated a dataset with 1831 training examples comprising of antibodies—antigen sequences and their corresponding IC_50_ values. The CATNAP output is comprised of site of antigen sequence alignment for each of the antibodies with respect to 2F5, 4E10 and 10E8. Using the co-crystalized structure of (2F5-ELDKWAS) in (PDB:1TJG)^34^ as template, the antibody fragment that comes in contact with the antigen was found by considering amino acids within 7 Å of the antigen in the co-crystallized structure.

To make the dataset more diverse and train a more robust ML model, we included more available antibody-antigen sequences and their neutralization potential. To do this, we compiled the sequences of Influenza, Dengue, Ebola, SARS, Hepatitis, etc.^30,37–90^ by searching the keywords of “virus, antibody” on RCSB server^91^ and selected the neutralizing complex by reading their corresponding publications. Furthermore, for each neutralizing complex, the contact residues at the interface of antibody and antigen were selected (Fig. S3). To select the antigen contact sequences, all amino acids within 5 Å of corresponding antibody were chosen (Figs. S4, S5). To select the antibody contact sequences, all amino acids within 5 Å of the antigen were chosen. In total, 102 sequences of antibody-antigen complexes were mined, comprising of structures from X-ray diffraction of crystal structure and Cryo-EM experiments, and added to the 1831 samples collected from CATNAP, resulting in total number of 1933 training samples.

### Graph featurization and machine learning

For effective representation of molecular structure of amino acids, the individual atoms of amino acids of antibody and antigen were treated as undirected graph, where the atoms are nodes and bonds are edges^92^. In this work, we generate the representations of molecules from their respective molecular graphs. We construct these molecular graphs using RDkit^93^. Embeddings are generated to encode relevant features about the molecular graph^94,95^. These embeddings encode information like the type of atom, valency of an atom, hybridization state, aromaticity etc. (Table S3). First, each antibody and antigen were encoded into separate embeddings and then concatenated into a single embedding for the entire antibody-antigen complex. We then apply mean pooling over the features for this concatenated embedding to ensure dimensional consistency across the training data. The pooled information is then passed to classifier algorithms like XGBoost^96^, Random Forest^97^, Multilayer perceptron, Support Vector Machine (SVM)^98^ and Logistic Regression which then predict whether the antibody is capable of neutralizing the virus. XGBoost is a gradient boosting framework which uses the second order derivative to approximate gradient to learn the features^96^. Random forest is an ensemble machine learning method as it uses multiple decision tress and selects the mode of these decision tress as the output^97^. Multilayer perceptron is a feedforward artificial neural network (ANN) which is composed of fully connected layers of perceptrons with an activation function. SVM is a machine learning algorithm which tries to learn the maximum-margin hyperplane to classify the data^98^. Logistic regression is the estimation of the parameters of a logistic model which is used to model the probability of different classes.

### Hypothetical antibody library generation

In order to find potential antibody candidates for SARS-CoV-2, 2589 different mutant strains of antibody sequences were generated based on the sequence of SARS neutralizing antibodies. The reason we selected these antibodies as initial scaffolds is that the genome of SARS-CoV-2^4^ is 79.8% identical to “Tor2” isolate of SARS (Accession number: AY274119)^99^. Exhaustive search of the RCSB PDB server concluded that 4 structures SARS (PDB: 2GHW, 3BGF, 6NB6, 2DD8) were the only SARS antigen and antibody complexes which have been reported till date. Using 4 different antibody variants of SARS^30,80,85,90^, point mutations were applied to all the amino acids in the binding region of antibody. (See “Supporting Information” for SARS-CoV-2 antigen and antibody interactions.) To find out the binding region of these antibodies for sequence generation, all amino acids within 5 Å of their respective antigen were chosen. To assess the biological feasibility of these mutant sequences, we scored each mutation by using the BLOSUM62 matrix^100^.

### Molecular dynamics simulations

To assess the stability of proposed antibody structures, we performed molecular dynamics (MD) simulations of each of antibody structure in a solvated environment^101^. The simulation of solvated antibody was carried out using GROMACS-5.1.4^102–104^, and topologies for each antibody were generated according the GROMOS 54a7^105^ forcefield. The protein was centered in a box, extending 1 nm from surface of the protein. This box was the solvated by the SPC216 model water atoms, pre-equilibrated at 300 K. The antibody system in general carried a net positive charge and it was neutralized by the counter ions. Energy minimization was carried out using steepest descent algorithm, while restraining the peptide backbone to remove the steric clashes in atoms and subsequently optimize solvent molecule geometry. The cut-off distance criteria for this minimization were forces less than 100.0 kJ/mol/nm or number of steps exceeding 50,000. This minimized structure was the sent to two rounds of equilibration at 300 K. First, an NVT ensemble for 50 picoseconds and a 2-femtosecond time step. Leapfrog dynamics integrator was used with Verlet scheme, neighbor-list was updated every ten steps. All the ensembles were under Periodic Boundary Conditions and harmonic constraints were applied by the LINCS algorithm^106^; under this scheme the long-range electrostatic interactions were computed by Particle Mesh Ewald (PME) algorithm^107^. Berendsen thermostat was used for temperature coupling and pressure coupling was done using the Parrinello-Rahman barostat^108,109^. The last round of NPT simulation ensures that the simulated system is at physiological temperature and pressure. The system volume was free to change in the NPT ensemble but in fact did not change significantly during the course of the simulation. Following the rounds of equilibration, production run for the system was carried out in NPT and no constraints for a total of 150 ns, under identical simulation parameters.

## Results and discussions

**Figure 1:**
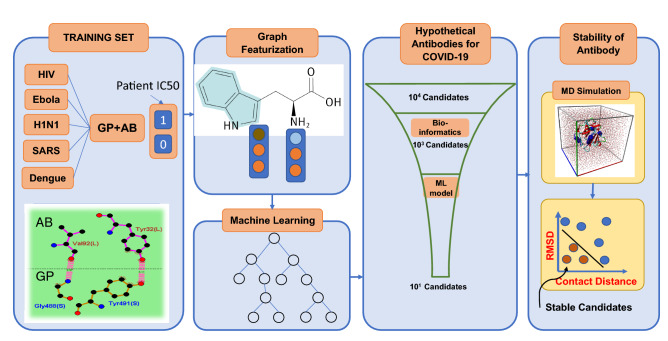
Designing antibodies or peptide sequences that can inhibit the SARS-CoV-2 virus requires high throughput experimentation of vastly mutated sequences of potential inhibitors. The screening of thousands of available strains of antibodies are prohibitively expensive, and not feasible due to lack of available structures. However, machine learning models can enable the rapid and inexpensive exploration of vast sequence space on the computer in a fraction of seconds. We collected 1933 virus-antibody sequences with clinical patient IC_50_ data. Graph featurization of antibody-antigen sequences creates a unique molecular representation. Using graph representation, we benchmarked and used a variety of shallow and deep learning models and selected XGBoost because of its superior performance and interpretability. We trained our model using a dataset including 1933 diverse virus epitope and the antibodies. To generate the hypothetical antibody library, we mutated the SARS scaffold antibody of 2006 (PDB:2GHW) and generated thousands of possible candidates. Using the ML model, we classified these sequences and selected the top 18 sequences that will neutralize SARS-CoV-2 with high confidence. We used MD simulations to check the stability of the 18 sequences and rank them based on their stability.

The flowchart of SARS-CoV-2 antibody discovery using ML has four major steps (Fig. 1): (1) collecting data and curating the dataset for training set. (2) Featurization, embedding and benchmarking ML models and selecting the best performing one. (3) Hypothetical antibody library generation and ML screening for neutralization and (4) checking the stability of proposed antibodies. This workflow enables the rapid screening of large space of potential antibodies to neutralize COVID-19. In general, this workflow can be used for high throughput screening of antibodies for any type of virus by only knowing the sequences of antigen epitopes.

**Figure 2:**
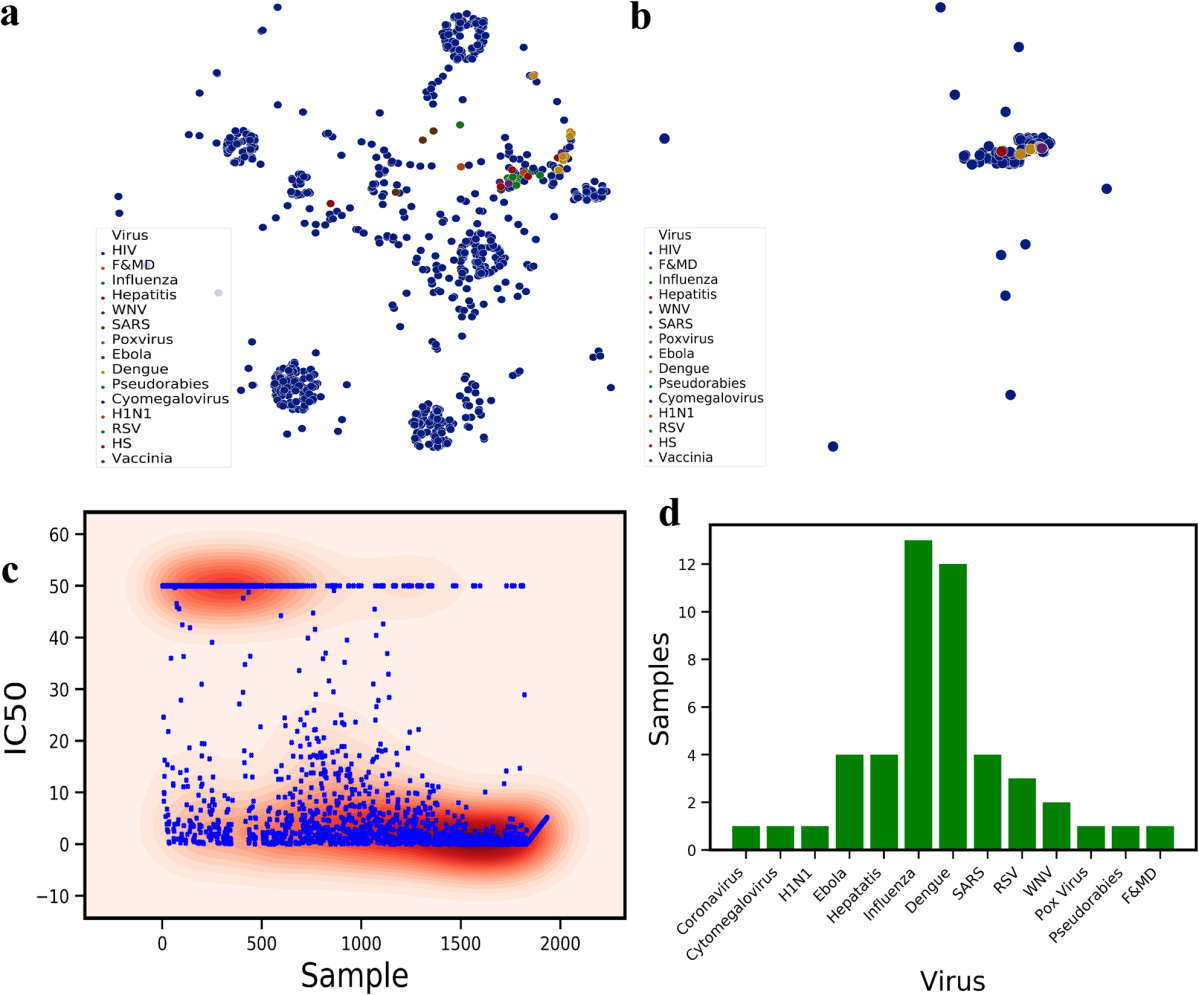
(**a**) t-Distributed Stochastic Neighbor Embedding (t-SNE) of all the viruses epitopes used in the training dataset, revealing biological similarity and diversity of the sequences used in the dataset. (**b**) t-SNE of all the therapeutics antibody sequences used in the training set for variety of different virus types. The majority of the broadly neutralizing antibodies such as 2F5 is clustered at the center of this plot. (**c**) Patient clinical IC50 data obtained from various sources and the distribution of the neutralizing (IC_50_ < 10) and Non-neutralizing (IC_50_ > 10) samples. (**d**) The number of samples for each virus class except HIV. For HIV, we collected 1883 samples. Influenza and Dengue has 10+ samples.

To better understand the diversity and similarity of the sequences that were used in the training set, we project the graph embeddings encoding the fingerprints of the molecules in the t-Distributed Stochastic Neighbor Embedding (t-SNE) space (Fig. 2a). t-SNE axes shows the directions of the maximum variance in the feature space of the dataset, therefore, the dimensionality of the data can be reduced to lower dimensions (here two). HIV antigen shows the most variations on t-SNE components where viruses such as Influenza, Dengue and H1N1 are very close to each other. The neutralizing antibodies were also projected using t-SNE to show the variations in the available neutralizing sequences (Fig. 2b). Unlike antigen variations, antibody sequences are much closer to the center of t-SNE with a few scattered ones. The comparison of Fig. 2a,b shows that the neutralizing antibodies are not sequence-diverse compared to virus antigens. This difference demonstrates that a large space of potential antibodies can be screened and used for finding novel antibodies. The labels in the dataset are comprised of the neutralization panel data, IC_50_ values for monoclonal antibodies and pseudo-typed viruses (Fig. 2c). The IC_50_ labels were collected from 49 published neutralization studies and were collected from CATNAP Database (for 1831 samples in our training set). For some cases in CATNAP, personal communication with the authors were made to resolve sequence name ambiguities between different laboratories. For 102 samples of various viruses collected from RCSB server, all of them neutralize their antigen based on biochemical assays. These samples were labeled by setting their IC_50_ to zero. Since classification is performed on the training dataset, IC_50_ ≤ 10 are set to neutralizing and IC_50_ > 10 to non-neutralizing (Fig. 2c). To visualize the diversity of the virus types used in the dataset other than HIV, the distribution of 13 more viruses were presented in Fig. 2d. Influenza, Dengue, SARS, Ebola and then Hepatitis have relatively larger samples in the dataset.

**Figure 3:**
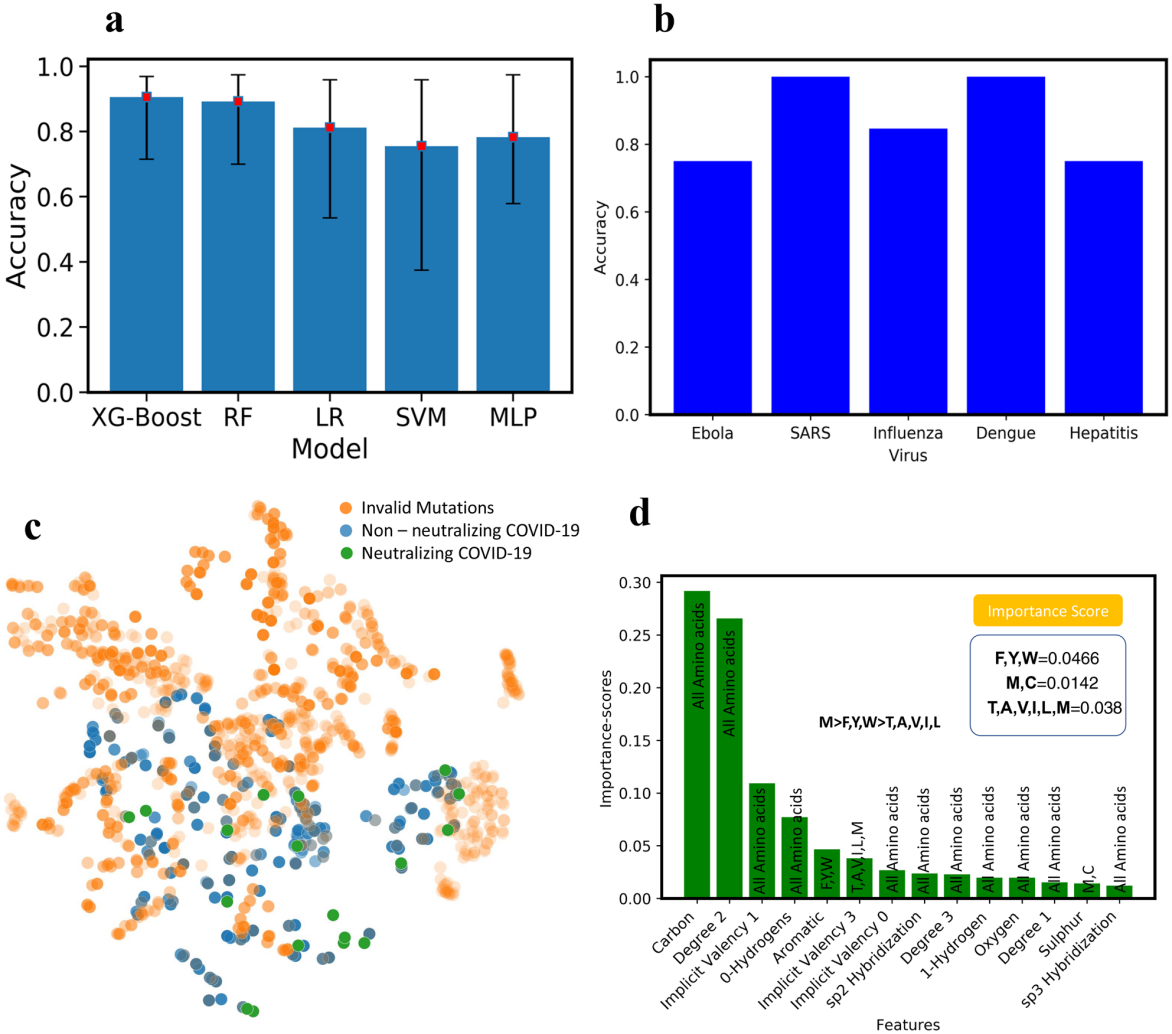
(**a**) The test accuracy with five-fold cross validation for XG-Boost, Random Forrest (RF), Logistic Regression (LR), Support Vector Machine (SVM) and Deep Learning (Multilayer Perceptron. XGBoost has the highest performance with (90.75%). (**b**) Out of training class test accuracy for influenza, Dengue, Ebola, Hepatitis, and SARS. To perform this test, for example for influenza, all the influenza virus-antibody sequences were removed from the training set and the obtained model were tested on all samples of Influenza and the accuracy is reported here. (**c**) Blosum validated mutations, non-neutralizing and neutralizing antibody sequences. To achieve more confidence, we set the threshold of prediction probability to 0.9895 in XGBoost and found 18 neutralizing antibody sequences (the green points). (**d**) Interpretability of ML model: to understand what mutations are playing the key roles in neutralization, XGBoost feature importance used with ranked atomic level features. Through connecting the atomic features with each of 20 amino acids, M was found to be the most important amino acids in neutralization followed by F, Y, W. The ML model predicted the presence of hydrophobicity and Sulfur as an important feature in antibody-antigen interaction. We concluded that M was the most important amino acid as it has both the characteristics of hydrophobicity and the presence of Sulfur.

To benchmark the performance of different ML models on the VirusNet dataset and select the best performing one, XGBoost, Random Forest (RF), Multilayer perceptron (MLP), Support Vector Machines (SVM), and Logistic Regression (LR) were used (Fig. 3a). The five-fold cross validation on 80–20% split, train, and test was implemented and best accuracy was observed for XG-Boost model. The performance and ranking of models follow the order of XGBoost (90.57%) > RF (89.18%) > LR (81.17%) > MLP (78.23) > SVM (75.49%). Since the featurized training data is sparse in the case of VirusNet (see Fig. 2a,b), XGBoost selects the sparse features input by pruning and learning the underlying sparsity patterns. In order to augment the accuracy as a performance metric we have also added ROC-AUC score as a performance metric in the “Supporting Information” (See Fig. S6). To test the robustness of the XGBoost on completely unseen virus types, for each left-out virus type, the model was trained on all the sequences in the VirusNet except for the left-out. For example, for Influenza, all the sequences and labels of Influenza were removed from the training set and the trained model on the remaining dataset were tested on all the Influenzas’s sequences and consequently the classification accuracy were reported (Fig. 3b). The accuracies for the out of class test is as follows: Influenza (84.61%), Dengue (100%), Ebola (75%), Hepatitis (75%), SARS (100%). From these results, we can conclude that our model performance will be reliable based on the accuracies for out-of-class prediction. The fact that our model prediction is highly accurate for various out of class tests, demonstrate its capability of effectively predicting the antibodies for novel SARS-CoV-2.

Next, using the best performing model (XGBoost), all the hypothetical candidates in the library were evaluated for neutralization. Out of all the candidates, some of them are invalid mutations screened using BLOSUM62 matrix^100^ (Fig. 3c). 18 final candidates are both valid mutations and can neutralize SARS-CoV-2 with high confidence probability of 0.9895 as per the ML model are then selected for MD screening (shown with green color in Fig. 3c).

A recent study reports that antibodies which effectively neutralized the previous SARS strains are not able to neutralize WH-Human 1^110^. However, the study also reports that there is “presence of a conserved immunogenic epitope among different Corona viruses”. Therefore, we had generated mutant and co-mutant sequences to create a diverse set of antibodies which we could screen through the ML model. The ML model we have developed uses the antigen–antibody interactions and tries to learn the structure-based mapping between the amino acids involved at interaction surface. This was the rationale behind including viruses from various other species as well in the dataset used to train the ML model. The dataset is sufficiently diverse so that the ML model can learn the structurally important features from variety of viruses, Fig. 2A, in addition to the sequence dependent information. This antigen diversity allowed us to overcome the constraint of dissimilarity in WH-Human 1 and SARS ACE2 receptor and yet make accurate predictions.

Interpretability of the ML models is very important in both explaining the underlying biological and chemical understanding of neutralization and providing design guidelines for antibody engineering. One of the significant advantages of ensemble methods such as XGBoost is their interpretability. By taking advantage of this property, the important features that are giving rise to neutralization were ranked and scored (Fig. 3d). The input features to the model contains atomic level attributes such as atom type, valency, hybridization, etc. To collectively translate the important atomic features into important amino acid features, the scores of amino acids with unique atomic features were summed up and ranked (Fig. 3d). Some of the atomic features were common among all the amino acids (e.g. Carbon, implicit valency, Oxygen, etc.) therefore; we ignored them. However, some of the other features like aromaticity or having Sulfur are unique and we considered those in amino acid features. Through this criteria some of the important mutations that we obtain include Cysteine, Methionine, Tyrosine, Phenylalanine and Tryptophan. Mutations to cysteine we concluded to not be viable as introduction of additional cysteine to the antibody structure, which heavily relies on disulfide bridges, would be detrimental as it can cause misfolding of the antibody structure. Further validation of this was done by the BLOSUM62 matrix, which put very heavy penalties on mutations which convert amino acids to cysteine. These observations cumulatively led us to the conclusion that Methionine is an important amino acid for antibody interface whereas cysteine is not. Methionine is known to be playing a crucial role in antigen recognition by antibody and further protein–protein interaction^111,112^. In addition, oxidative damage to Methionine is reported to have negatively impact the pharmacokinetic properties of antibodies^113^. This information validated the features learnt by the ML model, allowing us to definitively conclude that Methionine is indeed one of the important amino acids in antigen recognition by antibodies (Fig. 3d).

To validate the biological feasibility of the ML model predicted antibodies, we assessed the stability of the predicted antibody by Molecular Dynamics (MD) simulations. We assessed the antibodies based on two criteria's Root Mean Square Deviation (RMSD) and Mean Contact Distance^26^. RMSD is a measure of the deviation in the structure of the protein over the course of the simulation, a higher RMSD indicates that the structure is changing with respect to the initial structure for simulation. Contact Distance is the distance between the interacting amino acids of the protein, a higher Mean Contact Distance is indicative of an unstable protein as the amino acids are moving further apart. The combination of these features from simulation data of potentially neutralizing antibodies allowed us to validate their stability and select most stable candidates. The predicted sequences from the ML model were then used to model the novel structure of potentially neutralizing antibodies. The predicted sequences were projected onto their progenitor antibody and the changes in amino acid sequence were modelled as follows: simple point mutations were introduced by modifying the target amino acid using Coot^114,115^ (Crystallographic Object-Oriented Toolkit). Coot environment allowed us to predict the stereochemical effect of each point mutation and appropriately compensate for it. Using such an approach, we were able to accurately model the putative structures of the antibodies. The modelled structures were then passed to MD simulations for stability check.

**Figure 4:**
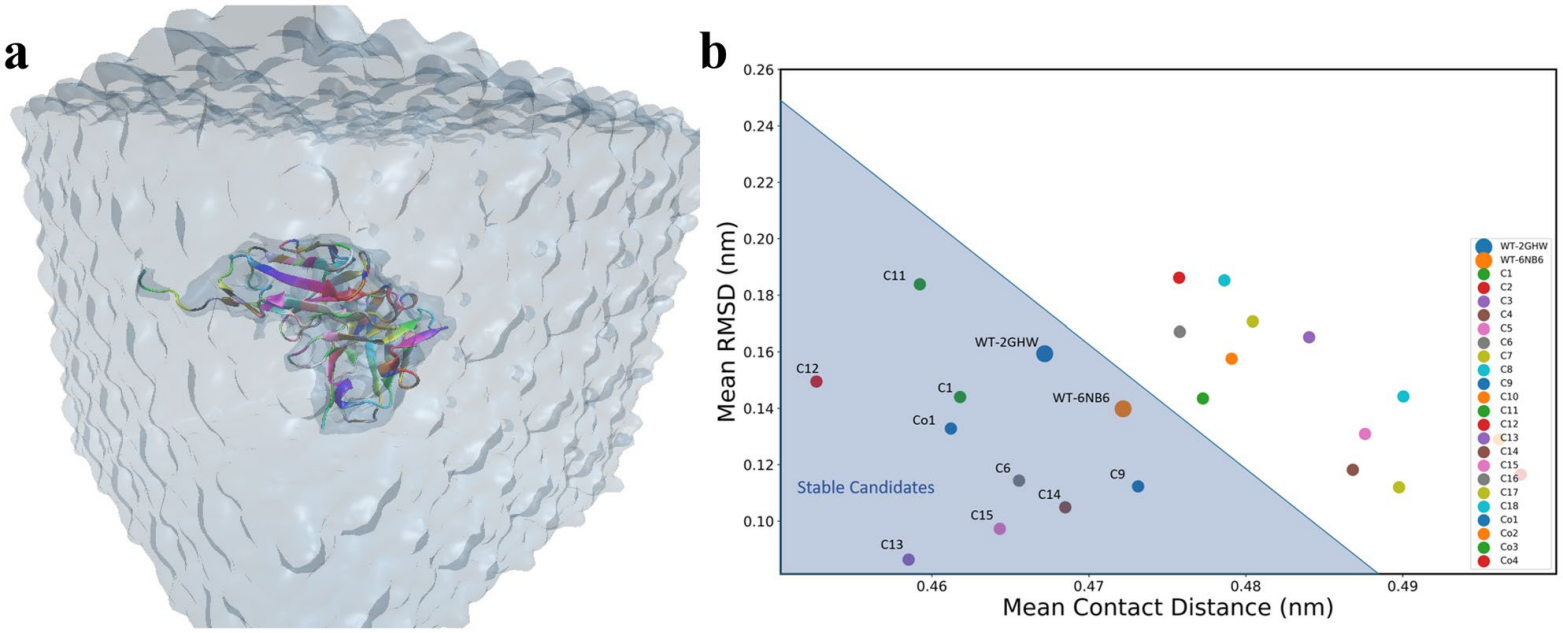
(**a**) The snapshot of MD simulation of mutated proteins. Each protein is solvated in a box of water and simulated to collect the statistical data on the stability of mutants and co-mutants. (**b**) Mean Root Mean Square Deviation (RMSD) versus Mean contact distances for each candidate averaged over the whole trajectory.

To check the stability of predicted structures energetically, 20 MD simulations (18 point mutations + 2 wildtype (WT)) in total were performed (Fig. 4a). Structures with low Root Mean Square Deviation (RMSD) and low contact distance are in a stable conformation, whereas structures with high RMSD and high contact distances are in an unstable conformation. RMSD (Fig. S2) and contact distance (Fig. S3) for WT structures have lower values, demonstrating stability, therefore; the contact distances versus RMSD is a good indicator of the stability of a protein over the course of a simulation (Fig. 4b).

Once mutation introduced in the crystallographic structure, it will cause it to deviate from WT structure’s RMSD and contact distance. We performed simulations for all the 18 point-mutant structures (Table S1) and their mean contact distance versus RMSD^116,117^ were computed for their respective trajectories (Fig. 4b) (see “Supporting Information”). Based on the two WT structures mean RMSD and contact distances, we selected the mutations which have mean contact distance and RMSD values less than 0.488 nm and 0.25 nm, respectively (the shaded triangle region in Fig. 4b). Candidates with higher values of mean RMSD and contact distances are unstable and will potentially fail to neutralize the SARS-CoV-2.

In order to be more comprehensive and take into account the effect of co-mutation, we created 5 co-mutations that are listed in Table S2 (Co1, Co2, Co3, Co4, Co5). These five co-mutations were screened using XGBoost for neutralization. Among all five co-mutations, Co5 did not neutralize. To check the stability of these four neutralizing co-mutations, MD simulations were performed and Co1 was found to be stable (Fig. 4b). The list of the final nine stable mutations and co-mutations are tabulated in Table 1 and the PDB structures are available in PDB format as “Supporting Information”.

**Table 1:** The final neutralizing candidates obtained through screening with ML model, MD simulation for stability and bioinformatics.

Structure	Mutation
C1	2GHW-A33C
C6	2GHW-R100H
C9	2GHW-R162H
C11	2GHW-T285N
C12	2GHW-R286H
C13	6NB6-F203M
C14	2GHW-T204N
C15	2GHW-T206N
Co1	6NB6-I51M, R150H, T204N

The detailed list of sequences is available in the “Supporting Information”.

## Conclusion

We have developed a machine learning model for high throughput screening of synthetic antibodies to discover antibodies that potentially inhibit SARS-CoV-2. Our approach can be widely applied to other viruses where only the sequences of viral coat protein-antibody pairs can be obtained. The ML models were trained on 14 different virus types and achieved over 90% fivefold test accuracy. The out of class prediction is 100% for SARS and 84.61% for Influenza, demonstrating the power of our model for neutralization prediction of antibodies for novel viruses like COVID-19. Using this model, the neutralization of thousands of hypothetical antibodies was predicted, and 18 antibodies were found to be highly efficient in neutralizing SARS-CoV-2. Using MD simulations, the stability of predicted antibodies were checked and nine stable antibodies were found that can neutralize SARS-CoV-2. In addition, the interpretation of ML model revealed that mutating to Methionine and Tyrosine is highly efficient in enhancing the affinity of antibodies to SARS-CoV-2. Further validation of the predicted antibodies can be carried out by future work involving in vitro experiments to assess the efficacy of the predicted antibodies at neutralizing the SARS-CoV-2 virus. In our work we assume only point mutations in the antibody sequence of SARS-CoV-1 when generating the potential antibody candidates for SARS-CoV-2, it is possible that the sequences have multiple point mutations and many different combinatorics. We would like to investigate such excluded combinations in the future and create a comprehensive dataset and a more robust protocol for discovering neutralizing antibodies.

## Supplementary Information

Supplementary Information 1.

Supplementary Information 2.

Supplementary Information 3.
