# Supplementary material for: Potential neutralizing antibodies discovered for novel corona virus using machine learning
Source: Sci Rep. 2021 Mar 4;11:5261. doi: 10.1038/s41598-021-84637-4 (PMC7970853; doi:10.1038/s41598-021-84637-4)
Supplement: Supplementary file 3 — Supplementary Information 3. [file 41598_2021_84637_MOESM3_ESM.docx]

Supporting Information Available

The RMSD and contact distance plots for all the trajectories versus time, the structure of virus antibody complex and the residues at the contact region, native contacts in antigen-antibody complex, the interaction of SARS-CoV-2 epitope with 2GHW antibody, tables for all neutralizing point mutations, co-mutations and their neutralization potentials, the structures of stable and unstable antibodies in PDB format, and IC50 data interpretation are available online. The VirusNet dataset can be found at https://github.com/BaratiLab/VirusNet.
